# Nanoscale Features of Tunable Bacterial Outer Membrane Models Revealed by Correlative Microscopy

**DOI:** 10.1021/acs.langmuir.2c00628

**Published:** 2022-06-24

**Authors:** Karan Bali, Zeinab Mohamed, Anna Scheeder, Anna-Maria Pappa, Susan Daniel, Clemens F. Kaminski, Róisín M. Owens, Ioanna Mela

**Affiliations:** † Department of Chemical Engineering and Biotechnology, University of Cambridge, Cambridge CB3 0AS, U.K.; ‡ School of Biomedical Engineering, Cornell University, Ithaca, New York 14853, United States; § Department of Biomedical Engineering, Khalifa University of Science and Technology, Abu Dhabi 127788, United Arab Emirates; ∥ School of Chemical and Biomolecular Engineering, Cornell University, Ithaca, New York 14853, United States

## Abstract

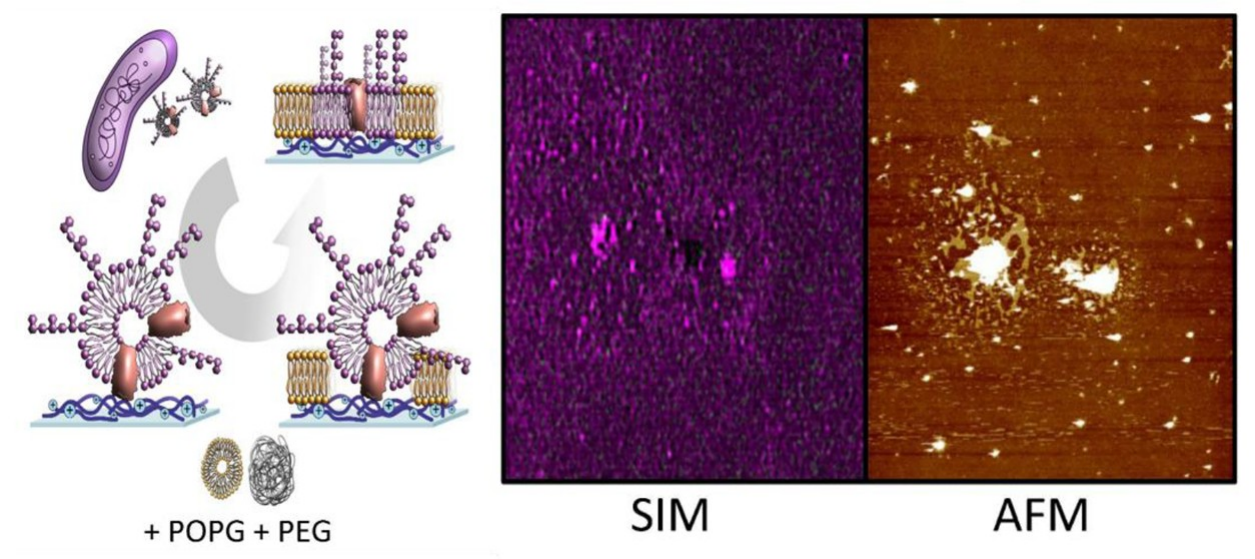

The rise of antibiotic resistance is a growing worldwide human health issue, with major socioeconomic implications. An understanding of the interactions occurring at the bacterial membrane is crucial for the generation of new antibiotics. Supported lipid bilayers (SLBs) made from reconstituted lipid vesicles have been used to mimic these membranes, but their utility has been restricted by the simplistic nature of these systems. A breakthrough in the field has come with the use of outer membrane vesicles derived from Gram-negative bacteria to form SLBs, thus providing a more physiologically relevant system. These complex bilayer systems hold promise but have not yet been fully characterized in terms of their composition, ratio of natural to synthetic components, and membrane protein content. Here, we use correlative atomic force microscopy (AFM) with structured illumination microscopy (SIM) for the accurate mapping of complex lipid bilayers that consist of a synthetic fraction and a fraction of lipids derived from *Escherichia coli* outer membrane vesicles (OMVs). We exploit the high resolution and molecular specificity that SIM can offer to identify areas of interest in these bilayers and the enhanced resolution that AFM provides to create detailed topography maps of the bilayers. We are thus able to understand the way in which the two different lipid fractions (natural and synthetic) mix within the bilayers, and we can quantify the amount of bacterial membrane incorporated into the bilayer. We prove the system’s tunability by generating bilayers made using OMVs engineered to contain a green fluorescent protein (GFP) binding nanobody fused with the porin OmpA. We are able to directly visualize protein–protein interactions between GFP and the nanobody complex. Our work sets the foundation for accurately understanding the composition and properties of OMV-derived SLBs to generate a high-resolution platform for investigating bacterial membrane interactions for the development of next-generation antibiotics.

## Introduction

In 2019, the World Health Organization declared that antimicrobial resistance is one of the 10 greatest threats to global health.^1^ A vast majority of antimicrobial-resistant strains of bacteria such as *Escherichia coli*, *Pseudomonas aeruginosa*, and *Acinetobacter baumanii* belong to the Gram-negative class of bacteria. Especially concerning is the prevalence of multidrug resistant strains of these bacteria; for instance, 17% of *E. coli* strains have been found to be multidrug resistant.^2^ In the search for novel strategies for combating these bacterial strains, it is crucial to precisely determine the events occurring at the level of the cell membrane, the outer membrane in the case of Gram-negative bacteria, the primary site of any interaction with the external environment. Reproduction of the *in vivo* cell membrane environment through the development of artificial lipid bilayers will reveal these crucial interactions.

Supported lipid bilayers (SLBs) made from reconstituted lipid vesicles are important tools in molecular biology, especially in the study of biological processes at the cellular or subcellular level. However, efforts to deepen our understanding of these processes in physiologically relevant environments are hampered by the simple nature of these bilayers. One approach to introducing physiologically relevant features into SLBs is the reconstitution of purified membrane proteins into proteoliposomes and the subsequent formation of SLBs from these proteoliposomes. This method has been used in several studies, including the study of protein–protein interactions,^3^ membrane–protein interactions and membrane remodeling,^4−6^ membrane poration,^7^ host–pathogen interactions,^8^ single-receptor activation,^9^ etc. Nevertheless, the method of purifying and reconstituting transmembrane proteins requires protein denaturing and refolding in the presence of detergents, which leads to low throughput and reproducibility issues. There is also a lack of control over protein orientation in the bilayers.^10^

A breakthrough in the field has come through the use of vesicles derived from cell membranes to form SLBs,^11^ and this can be applied specifically to creating Gram-negative outer membrane models. Gram-negative bacteria naturally produce vesicles that contain components of the outer membrane, known as outer membrane vesicles (OMVs). OMVs are 20–250 nm in diameter and are known to function in roles ranging from quorum sensing and signaling to horizontal gene transfer, all of which are important for bacterial communication and survival. OMVs can be easily isolated and harvested from bacteria through a series of centrifugation steps.^12^ By inducing OMVs to rupture and fuse with the addition of synthetic liposomes, one can generate outer membrane SLBs (OM-SLBs) that faithfully represent a naturally occurring outer membrane.^13^ These bilayers, which contain both bacterial and synthetic lipid fractions, have been shown to retain components such as outer membrane proteins and lipopolysaccharides in the correct orientation.^14^ Therefore, the system retains physiological properties that are beneficial to the investigation of protein–protein interactions, protein–ligand interactions, and other lipid membrane properties *in vitro*. Additionally, because these membrane models do not use live cells, they are much safer when investigating pathogenic bacterial membrane interactions. These complex bilayer systems hold promise but have not yet been fully characterized in terms of their composition and membrane protein content. Here, we use correlative microscopy to characterize the structural and functional properties of OM-SLBs at the nanoscale, in levels of detail that conventional microscopy methods cannot attain.

To visualize OM-SLBs, we combined atomic force microscopy (AFM) with structured illumination microscopy (SIM). AFM permits the direct observation of SLBs and proteins bound to them at high resolution (∼10 nm).^15^ However, AFM is a label-free technique and is thus incapable of identifying specific proteins of interest in lipid bilayers. Super-resolution microscopy techniques, such as SIM, enable visualization of specific molecules of interest through staining but at resolution lower than that of AFM.^16^ Here, we combine AFM with SIM to overcome the limitations of the two techniques and visualize OM-SLBs with high resolution and specificity.

## Experimental Section

### Growth of Bacterial Cultures and Isolation of OMVs

Five milliliters of liquid Luria-Bertani (LB) broth was inoculated with *E. coli* BL21(DE3) (Invitrogen) cells and grown for 16–20 h. Two milliliters of the overnight culture was added to 200 mL of LB broth and allowed to incubate at 37 °C for ∼3 h until the OD_600_ of the culture was ∼1.5. The culture was then centrifuged (4000*g*, 4 °C) for 15 min to remove cell debris, and the supernatant was collected. The supernatant was further passed through a 0.22 μm filter. The outer membrane vesicles (OMVs) were then isolated by ultracentrifugation (140000*g*, 4 °C) for 3 h (Beckman Coulter, type 50.2 Ti fixed-angle rotor), and the pellets were resuspended in 250 μL of phosphate-buffered saline (PBS) supplemented with a 2 mM MgCl_2_ solution. Finally, the OMV solution was centrifuged (16000*g*, 4 °C) for 30 min to remove any final contaminants such as flagella. The supernatant was collected and resuspended in 500 μL of PBS supplemented with a 2 mM MgCl_2_ solution. The final OMV solutions were then stored at −80 °C for further experiments.

### Preparation of Synthetic Liposomes

1-Palmitoyl-2-oleoyl-*sn*-glycero-3-phospho(1′-*rac*-glycerol) (POPG), purchased from Avanti Polar Lipids and stored in a chloroform solution at −20 °C, was used to prepare synthetic lipid liposomes. A nitrogen stream was used to evaporate the chloroform, and the sample was further desiccated for 1 h in a vacuum. The lipids were then hydrated in PBS supplemented with 2 mM MgCl_2_ to give a final lipid concentration of 4 mg/mL. Single unilamellar vesicles were made by lipid extrusion through a 50 nm pore polycarbonate membrane, and samples were stored for up to 2 weeks at 4 °C.

### Formation of Supported Lipid Bilayers on Glass Coverslips

Glass coverslips (Academy, 22 mm × 40 mm, 0.16–0.19 mm thick) were first cleaned with acetone and isopropanol before being functionalized by incubation with a poly-l-lysine solution [0.1% (w/v)] for 15 min. The PLL solution was washed away with deionized H_2_O before 100 μL of ∼10^10^ OMVs/mL was added to the glass slide. The OMVs were allowed to incubate for 20 min before being washed twice with a PBS solution to remove excess unadhered OMVs; 100 μL of the synthetic lipid vesicles was then added for 1 h to induce rupturing of the OMVs. The well was then washed again twice with PBS, and finally 30% PEG was added for 10 min as a final SLB formation step. The SLBs were then kept in a PBS solution for imaging. For experiments involving only synthetic lipid bilayers, the same protocol was followed without the first OMV incubation step.

### Characterization of SLBs Using Fluorescence Recovery after Photobleaching (FRAP)

Prior to analysis by fluorescence recovery after photobleaching (FRAP), OMVs must be fluorescently labeled. This was achieved by adding 1 μL of octadecyl rhodamine chloride-18 (R18) dye (Invitrogen) to 200 μL of the OMV solution and sonicating for 15 min. A G25 spin column (GE Healthcare) was used to remove unbound/excess R18 by centrifugation at 3000 rpm for 3 min at room temperature. Lipid bilayers were formed using the protocol outlined above.

FRAP measurements were conducted using an inverted Zeiss LSM800 confocal microscope with a 10× objective lens. A 30 μm diameter bleaching spot was made, and recovery of the fluorescence intensity of this spot was measured over time relative to a 50 μm diameter reference spot. The data were analyzed using MATLAB, and the fluorescence recovery was modeled using a modified Bessel function as described by Soumpasis et al.^17^ The model fit was used to extract the diffusion coefficient (*D*) according to the equation *D* = *r*^2^/4τ, where *r* is the radius of the photobleached spot and τ is the characteristic diffusion time. The fit was also used to extract the mobile fraction (MF) according to the terms (*I*_E_ – *I*_0_)/(*I*_I_ – *I*_0_), where *I*_E_ is the final postbleach intensity value, *I*_0_ is the first postbleach intensity value, and *I*_I_ is the initial prebleach intensity value.

### Production of OMVs Expressing the Lpp-OmpA-GFP Binding Nanobody

The pK:LppOmpA-NB plasmid was kindly provided by M. Norholm (Technical University of Denmark) and transformed into *E. coli* BL21(DE3) cells as described previously.^18^ To express the protein in OMVs, OMVs were grown in the same manner as described above except that 5 mM rhamnose was added to the bacterial culture at an OD of ∼0.3 to induce nanobody expression. The culture was then grown for a further 5 h after induction to ensure nanobody OMV production.

### GFP Binding Assays for OM SLBs

Bilayers were formed by the process described previously and incubated with a 0.06 mg/mL GFP solution (Sino Biological) for 30 min at 30 °C. The incubation was stopped by removing the GFP solution and washing with PBS buffer three times before imaging.

### Correlative AFM/SIM and Data Analysis

Correlative AFM–SIM imaging was performed by combining a Bioscope Resolve system (Bruker) with a custom-built SIM system. The piezo stage of the SIM microscope was removed from the inverted microscope frame, and the stage of the AFM system was used to drive both microscopes at the same time. An image of the setup is shown in Figure S5. The stage of the specific AFM system is designed so that the sample holder allows for optical detection of specimens from below, while the AFM scanning head can access the sample from above. The fields of view (FOVs) of the two microscopes were aligned so that the AFM probe was positioned in the middle of the FOV of the SIM microscope, by carefully moving the AFM stage using the alignment knobs. The final, fine alignment was achieved by using a bright-field image of the “shadow” of the AFM cantilever, taken with the SIM, to precisely align the AFM probe with the SIM lens (Figure S6).

To acquire structured illumination microscopy images, a 60×/1.2 NA water immersion lens (UPLSAPO 60XW, Olympus) focused the structured illumination pattern onto the sample, and the same lens was also used to capture the fluorescence emission light before imaging onto an sCMOS camera (C11440, Hamamatsu). The wavelengths used for excitation were 561 nm (OBIS 561, Coherent) for the lipid bilayers and 488 nm (iBEAM-SMART-488, Toptica) for the GFP. Images were acquired using customized SIM software described previously.^19^

AFM images were acquired in Scanasyst mode using ScanasystFluid+ probes (Bruker), with a nominal spring constant of 0.7 N m^–1^ and a resonant frequency of 150 kHz. Images were recorded at scan speeds of 1.5 Hz and tip–sample interaction forces between 200 and 300 pN. Large-scale images (20 μm × 20 μm) were used to register the AFM with the SIM FOVs, and small (2 μm × 2 μm) scans were performed to better resolve the morphology of the bilayers. Raw AFM images were first order fitted with reference to the lipid bilayer. Height measurements on the bilayers were performed by taking cross sections across different areas of interest, using the Nanoscope analysis software (Bruker).

For quantification of the bacterial component in the bilayers, AFM micrographs were converted into eight-bit images using Fiji (ImageJ) and thresholded to the height of the synthetic bilayer component. The area covered by the bacterial component (above the threshold) was calculated using the inbuilt area measurement tool in Fiji.

## Results and Discussion

**Figure 1:**
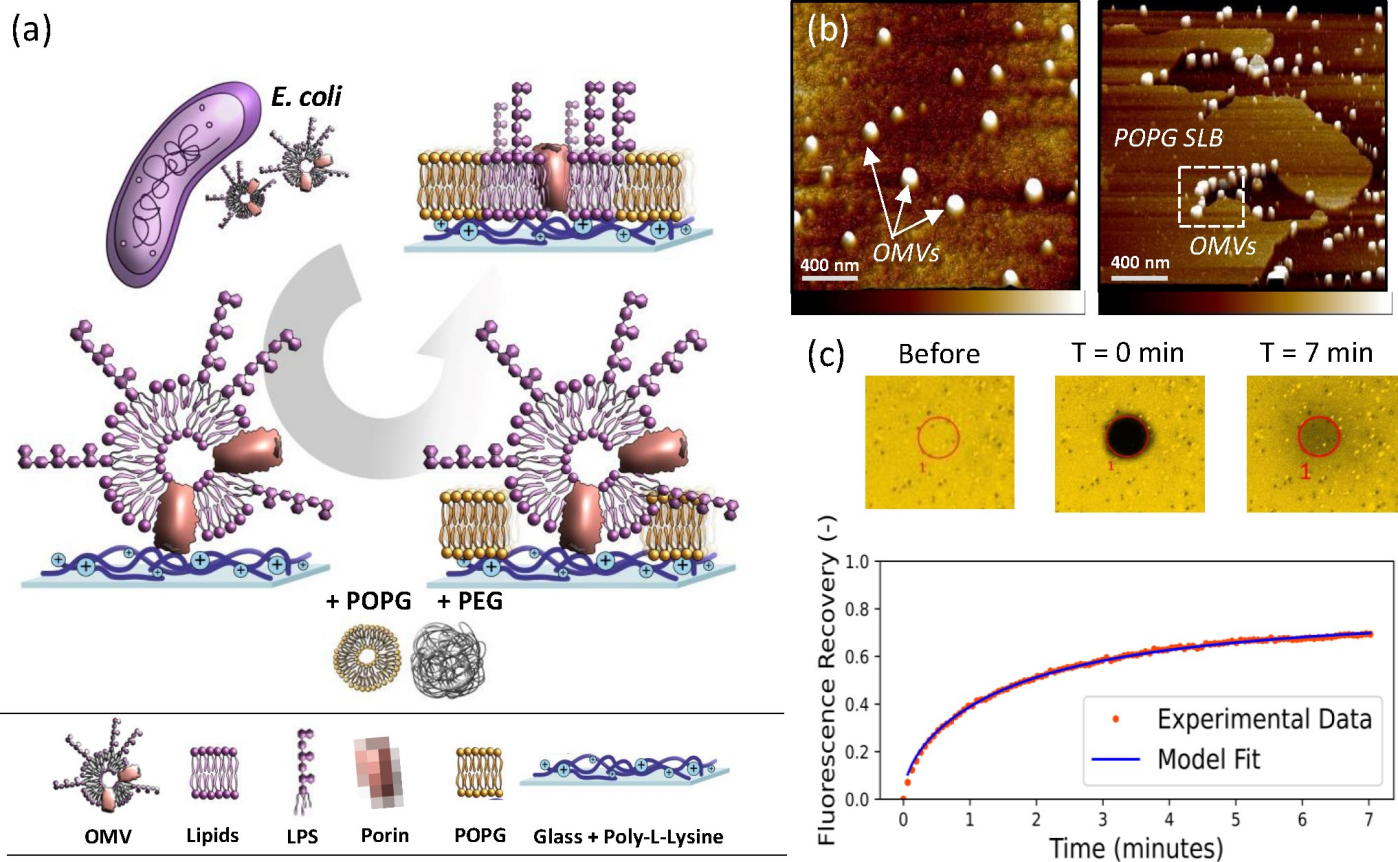
OM-SLB formation and fluorescence microscopy analysis. (a) Schematic showing the process of forming OM-SLBs on PLL-coated coverslips. OMVs are produced naturally by *E. coli* and harvested by ultracentrifugation. The OMVs (∼10^10^ particles/mL) are incubated on the coverslip surface before POPG and PEG are added sequentially to induce rupturing and fusing of the vesicles and the formation of a complete SLB. Naturally occurring membrane proteins are colored yellow. (b) Three-dimensional AFM images depicting different stages of the bilayer formation process (note that individual images depict different samples): intact OMVs on the PLL-coated glass surface before rupture (left) and an area of a partially formed bilayer (right). The synthetic POPG bilayer can be seen on the left side of the image, engulfing the OMVs and causing them to rupture. (c) FRAP data for the OM-SLB, showing that the bleached circle (diameter of 30 μm) recovers fluorescence over time. The diffusion coefficient (*D*) and mobile fraction (MF) values are 0.74 ± 0.14 μm^2^/s and 0.83 ± 0.06, respectively.

Our strategy for generating bacterial OMV-derived lipid bilayers is depicted in Figure 1a. We grew *E. coli* BL21(DE3) from an overnight culture and isolated OMVs as described in the Experimental Section. The dynamic light scattering (DLS) measurements showed an average hydrodynamic size of 101 ± 3 nm, which lies within the size range of OMVs. This was further confirmed by nanoparticle tracking analysis (NTA), a more precise method of size determination compared to DLS because DLS measures fluctuations in scattering intensity from a sample as a whole whereas NTA allows the diffusion of individual particles to be tracked.^20,21^ This method identified the main subpeaks at 88 and 152 nm (Figure S1). The concentration of vesicles measured with NTA was ∼10^11^ particles/mL. Transmission electron microscopy was used to directly visualize the OMVs and showed that the vesicles were spherical with diameters in the size range determined by DLS and NTA (Figure S1). Moreover, the vesicles retain the natural composition of the outer membrane, including outer membrane proteins that are involved in a variety of important processes. We confirmed the presence of the membrane protein OmpC, which is involved in the transport of antibiotics and small molecules across the membrane as well as acting as a binding site for the T4 bacteriophage,^22,23^ by a dot blot assay (Figure S2).

We used these OMVs to form SLBs on coverslips via vesicle fusion, as depicted in Figure 1a. Briefly, the negatively charged OMVs adhere to glass coated with positively charged PLL. The OMVs are induced to rupture and fuse by the addition of palmitoyloleoylphosphatidylglycerol (POPG) liposomes followed by incubation at room temperature for 1 h. Bilayers made of synthetic lipids form easily on the glass substrate, and then as the edges of these bilayers approach and engulf the OMVs, these vesicles rupture and fuse. The final stage is the addition of a PEG solution, which aids in the bilayer formation process by inducing rupture of any remaining vesicles through osmotic stress.^24^ AFM imaging (Figure 1b) shows the vesicle fusion process taking place, where the approaching edge of the synthetic bilayer induces the rupture of the OMVs. Finally, the OMVs were stained with the fluorescent lipid-intercalating dye octadecyl rhodamine-18 chloride (R18), and the presence of a complete and mobile bilayer was confirmed by fluorescence recovery after photobleaching (FRAP), as shown in Figure 1c. The two parameters used to quantify FRAP measurements are the diffusion coefficient (*D*), which is the mean squared displacement time of the diffusing lipids, and the mobile fraction (MF), which is the proportion of mobile lipids in the bilayer. For the OM-SLB, the D and MF values were 0.74 ± 0.14 μm^2^/s and 0.91 ± 0.06, respectively. These values are comparable to those measured in previous studies of SLBs on glass and corroborate the formation of a contiguous, mobile bilayer.^13^

**Figure 2:**
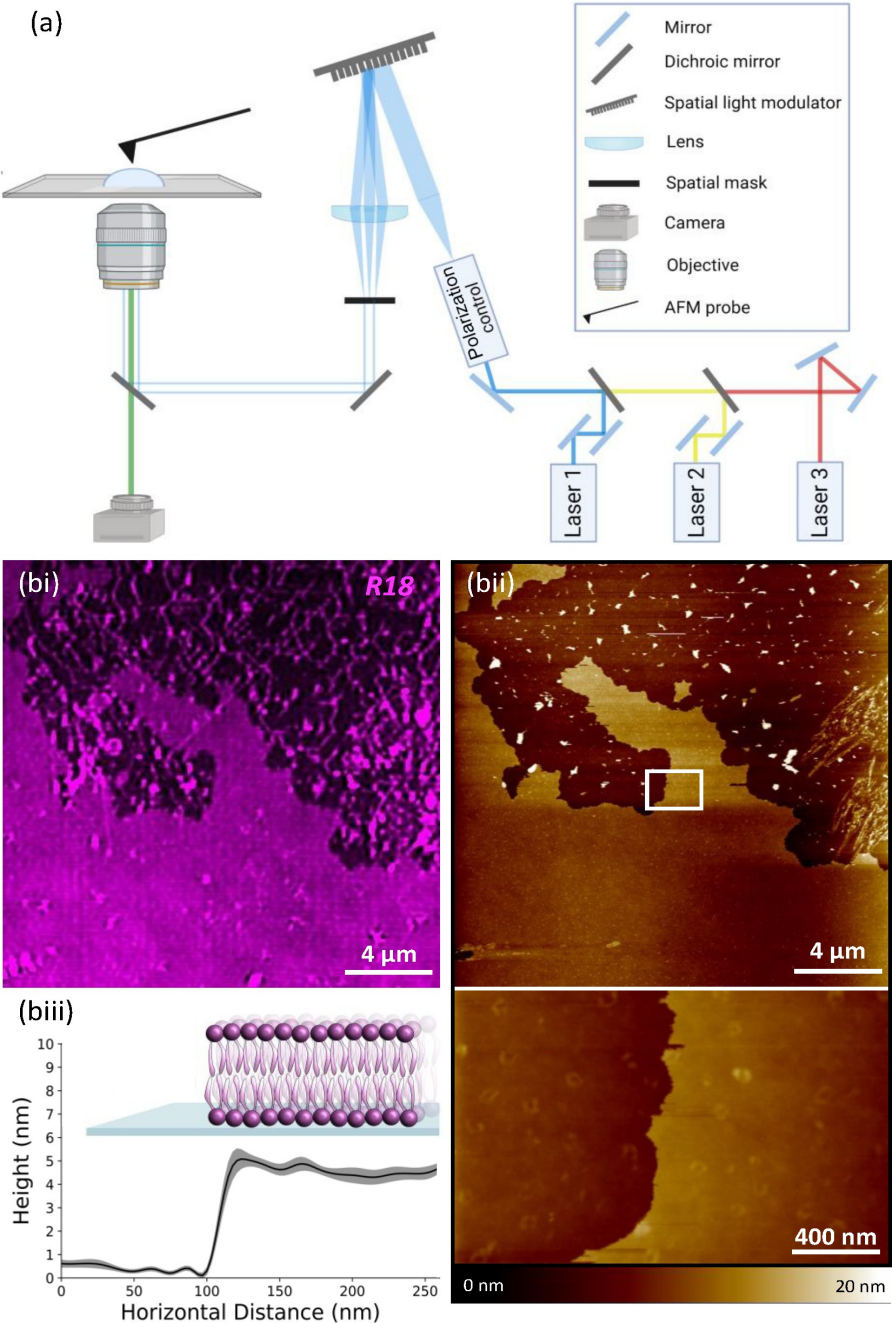
Correlative AFM/SIM imaging of a POPG SLB. (a) Simplified schematic of the correlative AFM/SIM microscope setup. (bi) Correlative imaging of a purely synthetic bilayer (POPG-SLB), formed by incubating 4 mg/mL POPG on a PLL-coated coverslip (left). The lipids are stained with R18 and visualized using SIM. (bii) The same area is imaged with AFM highlighting the ability of correlative microscopy to access precisely the same area of the bilayer. The bottom image shows a high-magnification area of the bilayer (represented by the white box in the original AFM image). The AFM height bar is 0–20 nm. (biii) Cross-sectional height analysis of the high-magnification AFM image and schematic representation of the POPG SLB formed on a glass substrate (not to scale). The height of the SLB is measured at ∼4 nm.

While confocal microscopy and FRAP characterization of the bilayers indicate the quality of the bilayers and enable quantification of the lipid mobility, the limited resolution of the technique does not allow for precise “mapping” of the synthetic and bacterial components of the OM-SLBs. Such mapping would enable, for example, quantification and optimization of the ratio of the natural to synthetic fraction in the bilayer, quantification of the membrane protein content, assessment of the distribution of the bacterial component within the full bilayer, and direct visualization of membrane–protein interactions.^25^ To enable such mapping, we used correlative AFM/SIM (Figure 2a) to sequentially visualize the same area with both techniques, as described in the Experimental Section.

We demonstrate the ability of the correlative system to image the same field of view using a POPG-only SLB sample, stained with R18 (Figure 2b). The edge of the bilayer is imaged because the distinctive shape helps to register the SIM and AFM images. Panels bi and bii of Figure 2 show the same SLB region imaged using SIM and AFM, respectively, and by zooming in on an area of the SLB with AFM, we obtain a highly precise level of topographical information about the bilayer, showing the bilayer to be smooth with few defects as would be expected from a synthetic bilayer. Furthermore, by taking five cross sections of ∼250 nm each of the SLB, we show that the average height of the bilayer is 4.4 ± 0.3 nm, which corresponds to the height of a synthetic lipid bilayer.^26,27^

**Figure 3:**
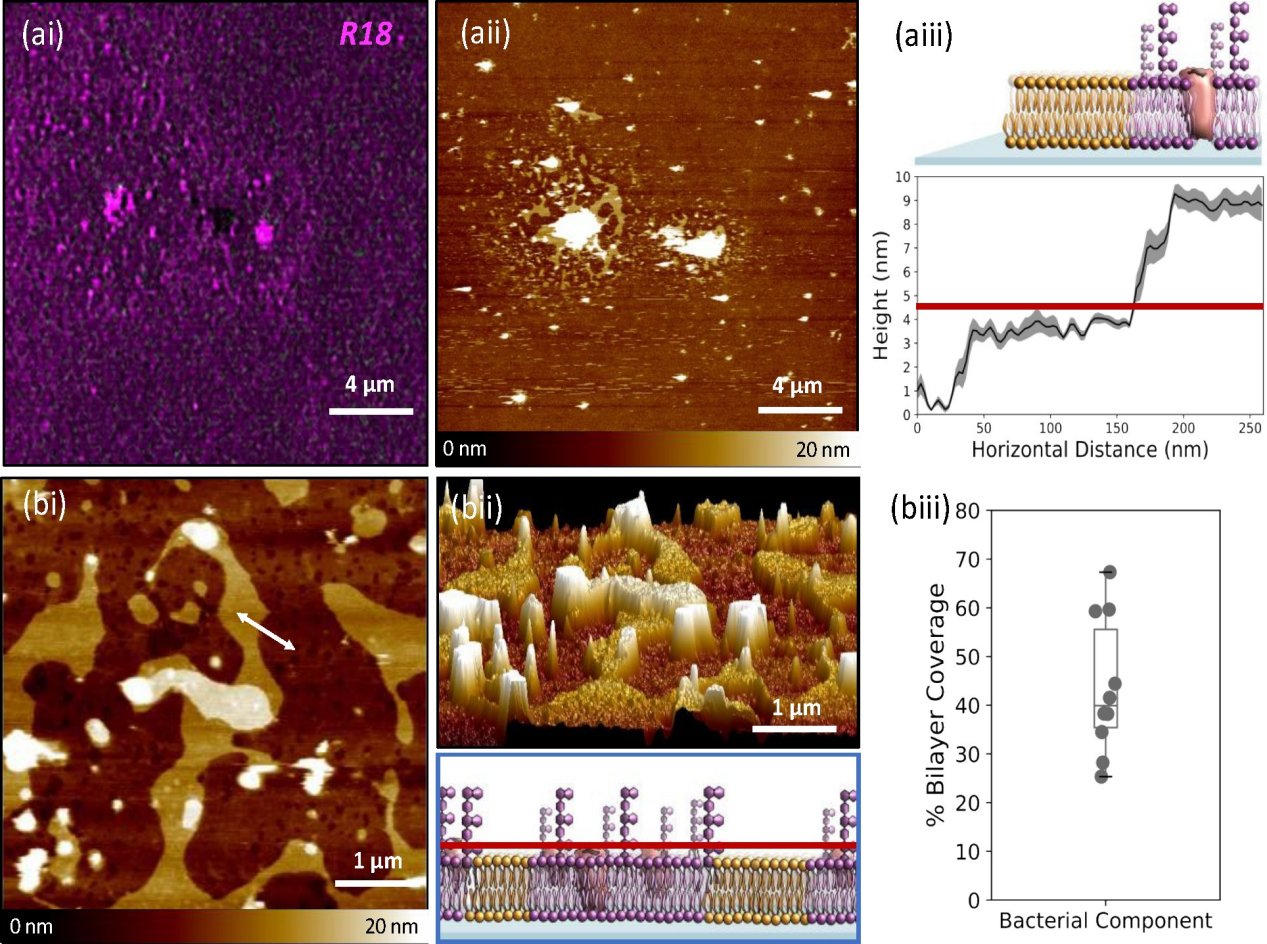
Imaging OM-SLBs using correlative AFM/SIM. (a) Correlative imaging of an OM-SLB, formed in the same manner as depicted in Figure 1. (ai) The OMVs are stained with R18, and the bilayer is imaged using SIM. (aii) The same area is imaged with AFM. Two distinct regions can be seen in the bilayer: a highly fluorescent region that corresponds to taller features in the AFM image and one exhibiting lower fluorescence levels where corresponding heights measured by AFM are ∼4 nm. (aiii) Cross-sectional height analysis (an average of five representative cross sections taken) shows the two regions of the bilayer correspond to heights of ∼4 and ∼9 nm. These represent the synthetic and bacterial component regions of the bilayer, respectively, in line with bilayer heights reported in the literature. The red line represents the threshold value above which exists the bacterial component of the bilayer. (bi) 5 μm × 5 μm AFM image of the OMSLB. The white arrow shows a representative section used for the cross sectional height analysis. AFM heights are 0–20 nm. (bii) Corresponding three-dimensional image that shows the areas of different heights corresponding to the synthetic and bacterial component regions (top). Schematic showing the bacterial and synthetic components of the bilayer, with the red line depicting the threshold used to calculate the areas of bacterial component as a proportion of total bilayer coverage (bottom). (biii) Quantification of the bacterial component as a percentage of the entire SLB imaging area. The box and whisker plot represents the following percentiles: minimum, 25.3%; 25th percentile, 35.4%; 50th percentile, 39.9%; 75th percentile, 55.6%; maximum, 67.3%.

Having established the power of the correlative microscopy method to track precisely the same area of the bilayer, we move on to imaging SLBs that contain OMVs as depicted in Figure 1. In this case, only the OMVs were stained with R18, while the synthetic POPG lipids were unstained to enable distinction of the two fractions (Figure 3). When the resulting OM-SLB was assessed with SIM, areas of high and low fluorescence were seen throughout the bilayer, indicating diffusion of phospholipids between the two fractions and demonstrating that the bilayer is complete and continuous. Because the fluorescence from the R18 dye is present in both bilayer regions even though only the OMVs were initially stained, we speculate that this could be attributed to the ability of phospholipids to diffuse between the two regions. The areas of high R18 fluorescence could then arise due to the presence of lipopolysaccharide (LPS) in the OM-SLB regions hindering R18 diffusion because the LPS in the outer leaflet of the OM-SLB diffuses much more slowly, as has been suggested by molecular dynamics simulations.^28^ Therefore, areas of high fluorescence were interpreted as originating from OMVs and used as a beacon for the bacterial fraction within the bilayer.

When the same area was imaged using AFM, we saw that the bilayer was not smooth as was the case for the POPG bilayer, but there were distinct bilayer regions with different heights that corresponded to areas of low and high fluorescence. We postulate that the areas of high fluorescence/height correspond to areas of OM from the *E. coli* cells while the low fluorescence/height regions correspond to areas of POPG SLB. Because the bilayer formation process is based on the synthetic bilayer forming first and then engulfing OMVs, causing them to fuse and rupture to form bacterial membrane patches, we hypothesize that the very high regions at the center of these patches correspond to still unruptured OMVs. The size of each bacterial patch is dependent on the number of OMVs that have been fused together (Figure S7). On the basis of this hypothesis, we quantified the area of the OM-SLB and POPG-SLB patches within the hybrid SLBs in 10 different regions and showed that the bacterial component covers 43 ± 14% of the total bilayer area. A cross-sectional height analysis, performed on multiple areas of the AFM images, revealed the lower height bilayer region, which corresponded to areas of low fluorescence, to be 3.75 ± 0.27 nm, suggesting this is POPG SLB. The higher SLB patches, which corresponded to areas of high fluorescence, were 8.75 ± 0.13 nm in height. This height corresponds to the reported height of the OM in *E. coli* cells,^29^ which is greater than the height of a synthetic lipid bilayer, because of the presence of lipopolysaccharides. The combination of SIM and AFM, therefore, reveals that the hybrid SLB contains discrete patches of OM-SLB and POPG-SLB. We theorize that this lack of mixing may be overcome by the use of a cushioned substrate upon which to generate SLBs, such as the conducting polymer PEDOT:PSS.

**Figure 4:**
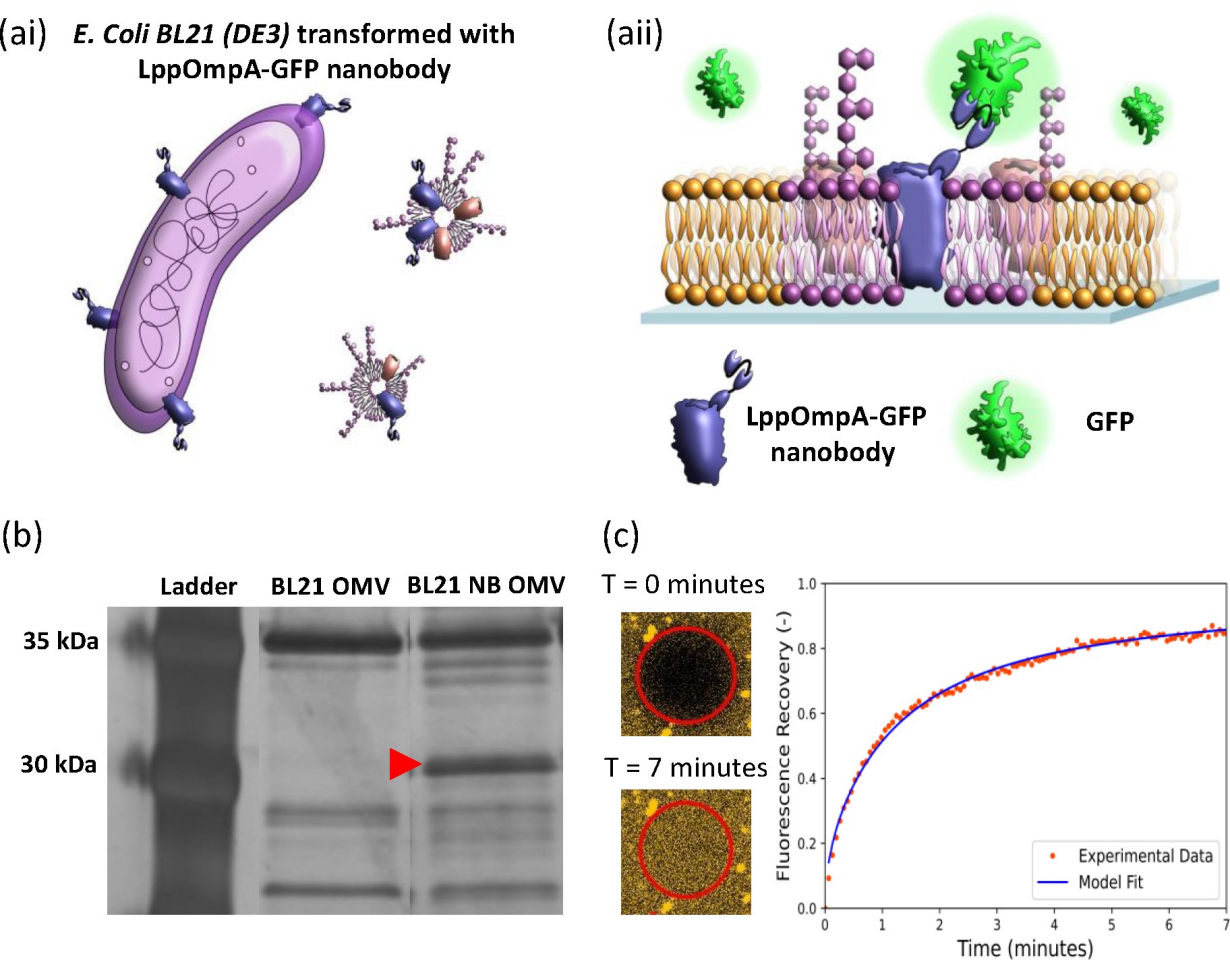
(ai) Schematic showing OMVs expressing the nanobody complex produced from transformed *E. coli* cells. (aii) Schematic of the OM-SLB formed using OMVs expressing the GFP binding nanobody complex. (b) SDS–PAGE gel of untransformed and transformed OMVs. The left lane shows the protein content of BL21(DE3) OMVs, while the right lane shows the protein content of the LppOmpA-GFP nanobody complex-transformed OMVs. A red arrow indicates the nanobody band, which has a molecular weight of ∼28 kDa. (c) FRAP data for the OM–nanobody SLB (diameter of bleached circle, 30 μm). The corresponding FRAP parameters are *D* = 0.94 ± 0.08 μm^2^/s and MF = 0.86 ± 0.10 for this bilayer.

One of the main strengths of the OM-SLB platform is the potential to easily express proteins of interest in the bacteria from which the OMVs are derived and, therefore, have these proteins present in the OMVs (Figure 4a). We demonstrated this ability by expressing a lipoprotein–outer membrane protein A–GFP binding nanobody (LppOmpA-GFP, hereafter called the nanobody complex) that specifically binds GFP.^18^ Using a GFP binding assay, we showed that the nanobody complex was expressed in BL21(DE3) cells (Figure S3). We isolated OMVs from the engineered *E. coli* cells as described above, with an extra rhamnose addition step to induce the expression of the nanobody complex, and ran both OMV types on an sodium dodecyl sulfate–polyacrylamide gel electrophoresis (SDS–PAGE) gel to compare the protein content (Figure 4b). An extra protein band was visible in the nanobody OMVs compared with the untransformed OMVs and corresponded to the expected molecular weight of the nanobody (∼28 kDa), indicating successful expression of the protein of interest in the OMVs. By following the same SLB formation sequence as in Figure 1a, we used the nanobody OMVs to make OM-SLBs containing the nanobody complex. FRAP confirmed the presence of a complete and mobile bilayer with the SLB showing a diffusion coefficient of 0.94 ± 0.08 μm^2^/s and a mobile fraction of 0.86 ± 0.10 (Figure 4c).

**Figure 5:**
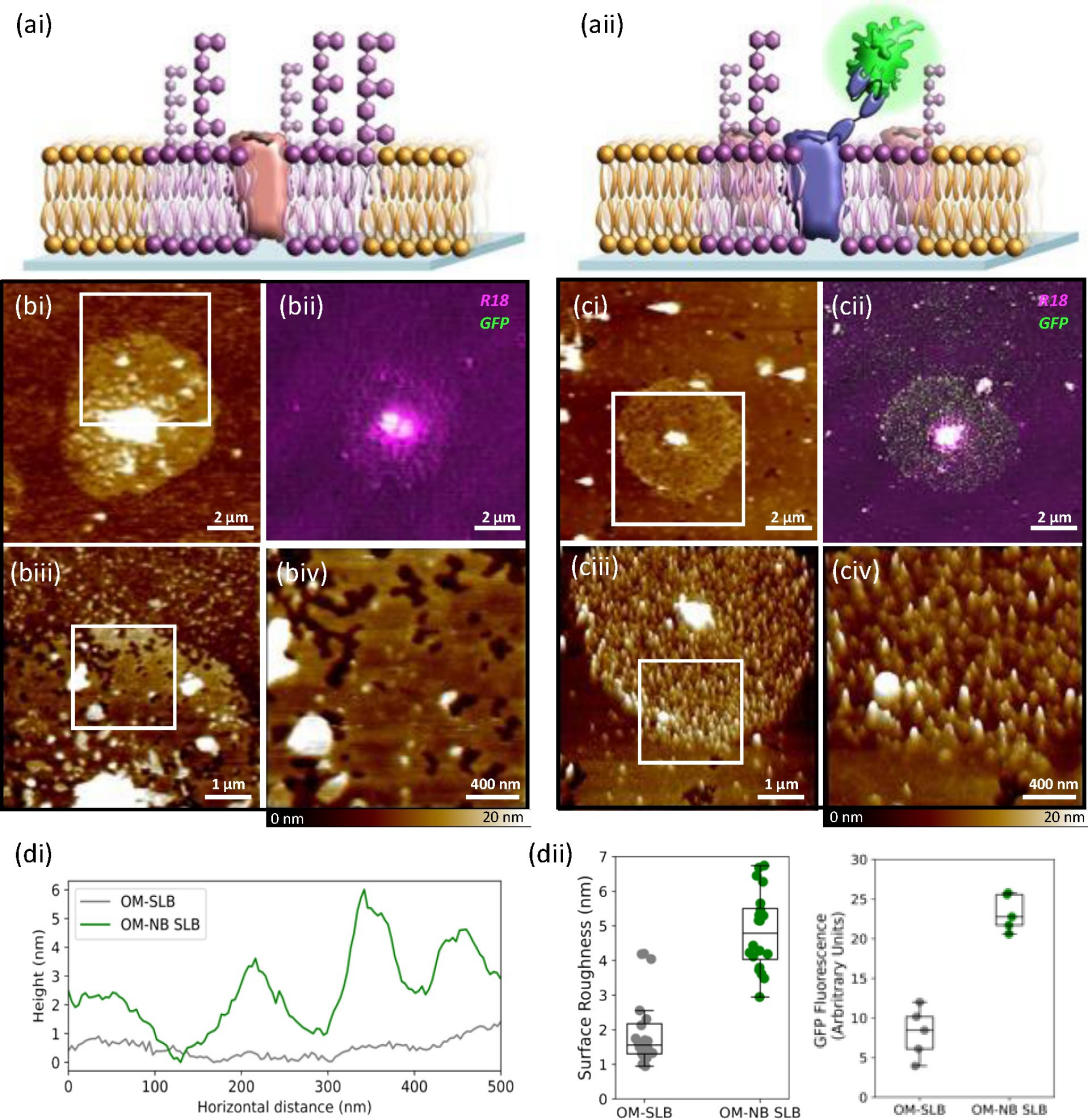
Schematic of (ai) OM-SLB and (aii) OM-SLB expressing the GFP binding nanobody complex in both cases after incubation with GFP. (bi) AFM image of a 10 μm × 10 μm area of the OMSLB. (bii) Corresponding reconstructed SIM image. (biii) 5 μm × 5 μm and (biv) 2 μm × 2 μm higher-magnification three-dimensional (3D) images of the bacterial component of the SLB. (ci) AFM image of a 10 μm × 10 μm area of the OM-NB SLB. (cii) Corresponding reconstructed SIM image. (ciii) 5 μm × 5 μm and (civ) 2 μm × 2 μm higher-magnification 3D images of the bacterial component of the OM-NB SLB. The height bar for each AFM image is 0–20 nm. (di) Height profile for a representative cross section of the OM-SLB vs OM-NB SLB, showing the increased height range in the case of the GFP-bound SLB. (dii) Surface roughness analysis for the OM and OM-NB SLBs after GFP incubation (left). The average surface roughness in the OM-NB SLB case is 4.85 nm, compared to just 1.94 nm for the OM SLB case. GFP fluorescence intensity signal for the OM-SLB vs the OM-NB SLB, showing the increase in the intensity of the fluorescence signal for the nanobody SLB (right).

Having established that the bacterial membrane forms discrete patches within the hybrid SLB, we used correlative AFM/SIM to visualize the localization of a specific protein within the bacterial component of the SLBs. We generated standard OM-SLBs and OM-SLBs from bacteria that expressed the nanobody complex (OM-NB SLB) and incubated both with GFP (Figure 5a). As seen previously, the OM-SLB shows two SLB regions of distinct height differences (Figure 5b). These regions are also evident in the case of the nanobody containing SLB, but crucially here the SIM reconstruction reveals the patches of bacterial SLB fluoresce strongly in the 488 nm wavelength region (Figure 5c), suggesting the presence of bound GFP. Furthermore, the correlative AFM images of the bilayers indicate the presence of surface features on the bacterial component of the nanobody bilayer that are not present in the control OM-SLB. This further confirms the existence of bacterial fractions within the bilayer, as the GFP can bind only to the nanobody complex that is present in the OMVs. A cross sectional analysis of the bacterial component of the OM-SLBs shows a range of 1.42 nm compared to a range of 6.01 nm for the OM-NB SLB (Figure 5di). A more in-depth analysis of the surface roughness at the bacterial fractions of the SLBs (Figure 5dii) showed that the mean height of the surface features in the OM NB-SLB with GFP bound was 4.85 nm (range of 2.95–6.74 nm), with this height corresponding to the reported length of a GFP molecule.^30^ By contrast, surface features in the control OM-SLB had a mean height of only 1.94 nm (range of 0.94–4.20 nm), and although there are outlier points in the OM-SLB roughness analysis, these likely reflect the presence of naturally occurring outer membrane proteins. Additionally, we quantified the difference in fluorescence between bacterial membrane patches that contain the nanobody complex and those that do not by calculating the corrected total green fluorescence (CTF) and showed that the average CTF for the OM–nanobody SLB is approximately double that of the OM-SLB (Figure 5dii) and that of a POPG SLB (Figure S4). The ability to map the bacterial component of the SLB using GFP binding is a key finding, as it shows that we can identify areas of interest in these complex systems using correlative AFM-SIM and quantify binding events occurring on these membranes. Furthermore, we can manipulate these systems precisely by altering the expression profile of the bacterial component. This is particularly exciting when we consider the future applications of this method, particularly with respect to antimicrobial screening studies.^14^ For instance, if we wish to analyze the interaction of a certain class of antibiotic with a protein target of interest in a bacterial membrane, we can overexpress or indeed delete this protein from our membranes. In this way, we have a platform for tailored pharmacological studies in a reproducible and safe to use manner. Moreover, we can combine microscopy with other *in vitro* techniques, such as electrical impedance spectroscopy, to further investigate the pharmacological profile of a given antimicrobial.

## Conclusions

In conclusion, we present here correlative AFM/SIM as a method for accurate characterization of bacterial supported lipid bilayers at the nanoscale. This approach enables not only the mapping and quantification of the bacterial and synthetic components within those bilayers but also the visualization of single proteins bound to those components. Having access to detailed maps of the bacterial and synthetic components of these bilayers enables optimization of these systems with respect to the quality of the bilayers and the quantity of the bacterial fraction. Our method has the potential to facilitate antimicrobial discovery by enabling investigation of how antimicrobial drugs and drug delivery vehicles, such as nanomaterials and bacteriophages, interact with the bacterial membrane. In this study, we have optimized our method for visualization of SLBs that contain bacterial membrane fractions, but correlative AFM/SIM can be employed to characterize SLBs that contain natural components derived from mammalian cells, plants, and other organisms. Furthermore, the method can be adapted for a variety of other applications, ranging from investigation of single-molecule interactions to characterization of inorganic two-dimensional materials.

## Supporting Information Available

Additional experimental details, materials, and methods, including photographs of the experimental setup (PDF)
