# Supplementary material for: Nanoscale Features of Tunable Bacterial Outer Membrane Models Revealed by Correlative Microscopy
Source: Langmuir. 2022 Jun 24;38(29):8773–82. doi: 10.1021/acs.langmuir.2c00628 (PMC9330759; doi:10.1021/acs.langmuir.2c00628)
Supplement: Supplementary file 1 — la2c00628_si_001.pdf [file la2c00628_si_001.pdf]

Nanoscale features of tunable bacterial outer membrane models revealed by correlative microscopy

Karan Bali[a], Zeinab Mohamed[b] Anna Scheeder[a], Anna-Maria Pappa[c], Susan Daniel[b,d], Clemens F. Kaminski[a], Róisín M. Owens*[a], Ioanna Mela*[a]*

[a] Department of Chemical Engineering and Biotechnology, University of Cambridge, Cambridge, CB3 0AS, UK

[b] School of Biomedical Engineering, Cornell University, Ithaca, NY, 14853, USA

[c] Department of Biomedical Engineering, Khalifa University of Science and Technology, Abu Dhabi, 127788, United Arab Emirates

[d] School of Chemical and Biomolecular Engineering, Cornell University, Ithaca, NY, 14853, USA

Supplementary Material

Supplementary methods

<u>Characterization of OMVs</u>

1. Dynamic Light Scattering

Dynamic Light Scattering (DLS) measurements were performed using a Zetasizer Nano S90 (Malvern Panalytical) configured with a 633 nm laser and a 90º scattering optic. 1 ml of sample was transferred into a disposable plastic cuvette, and three runs were taken for each measurement. The intensity of the scattered light is used by the Zetasizer software to determine three main parameters: Z average (d.nm) which measures the average size of the particle distribution; count rate (kcps) which counts the number of photons detected per second and is related to the concentration and quality of the sample; polydispersity index (PDI) which provides a measure for the heterogeneity of the particle size distribution.

2. Nanoparticle Tracking Analysis

Nanoparticle Tracking Analysis (NTA) was carried out at the University of Cambridge Veterinary School. Samples were analyzed using a Nanosight NS500 (Malvern Panalytical) fitted with an Electron Multiplying Charged Couple Device (EMCCD) camera configured with a 522 nm laser. Prior to analysis, samples were diluted (1:500) in PBS. 5 x 60 second videos were recorded for each sample analyzed, with a temperature range of 20.8 – 21.5ºC and a camera level of 15. NTA 3.2 software was used to analyze the data with a detection threshold of 5.

3. Transmission Electron Microscopy

Transmission Electron Microscopy (TEM) was carried out at the Cambridge Advanced Imaging Centre. 10 μl of sample was negatively stained with 1% (w/v) uranyl acetate solution for 2 minutes at room temperature before being visualized with a Tecnai G2 80-200 keV transmission electron microscope, operating at 200 keV with images recorded with a bottom-mounted AMT CCD camera.

GFP binding assays for E. coli BL21 cells

Cells were pelleted from an overnight culture via centrifugation for 4 minutes at 2272xg, and resuspended in 50 µl of 50 mM Tris buffer, before being mixed with 50 µl of 0.12 mg/ml GFP solution (Sino Biological) and incubated for 30 minutes at 30ºC at 250-300 rpm. The incubation was stopped by centrifugating the cells for 4 minutes at 2272xg, and then the cells were washed twice with 300 µl 50 mM Tris buffer and resuspended in 200 µl 50 mM Tris buffer for imaging.

SDS-PAGE analysis of OMVs

OMVs were boiled at 95ºC for 5 minutes with lithium dodecyl sulfate (LDS) sample buffer (4X Bolt; Invitrogen) and run on a NuPAGE 12% Bis-Tris gel (Invitrogen) in MOPS buffer at 200 V for 30 minutes. The protein bands were visualized using a ProteoSilver Silver Stain kit (Sigma-Aldrich).

Dot Blot against OmpC

OMVs and POPG (used as a negative control) were targeted with OmpC monoclonal antibody (Bioorbyt). Goat Anti-Mouse IgG/ Horseradish Peroxidase Conjugate (HRP) (Novex, Life Technologies) was used as the secondary antibody. A PVDF membrane (ThermoFisher Scientific) with 0.45 µm pore size was activated by shortly immersing it in methanol, before being incubated in transfer buffer (Novex, Life Technologies) at room temperature for 3 minutes. The membrane was transferred onto a filter paper (ThermoFisher Scientific), previously soaked in transfer buffer, and 10 µL of each sample were added onto the membrane. The membrane was incubated in blocking buffer (0.1 % Tween 20 (ThermoFisher Scientific) and 5 % BSA (ThermoFisher Scientific) in PBS) at room temperature for 1hour to block unspecific sites, then it was incubated in primary antibody solution (1:1000 dilution in blocking buffer) at room temperature for 1 hour. The membrane was washed three times in washing buffer (0.1 % Tween 20 in PBS) at room temperature for 2 minutes, then incubated with secondary antibody solution (1:1000 dilution in washing buffer) at room temperature for 1 hour. The membrane was washed three times in washing buffer at room temperature for 2 minutes, before being transferred to a transparent film. After addition of the chemiluminescent solution (SuperSignal West Pico PLUS, ThermoFisher), the membrane was transferred to a black background for imaging (G:BOX mini 6/9, Syngene).

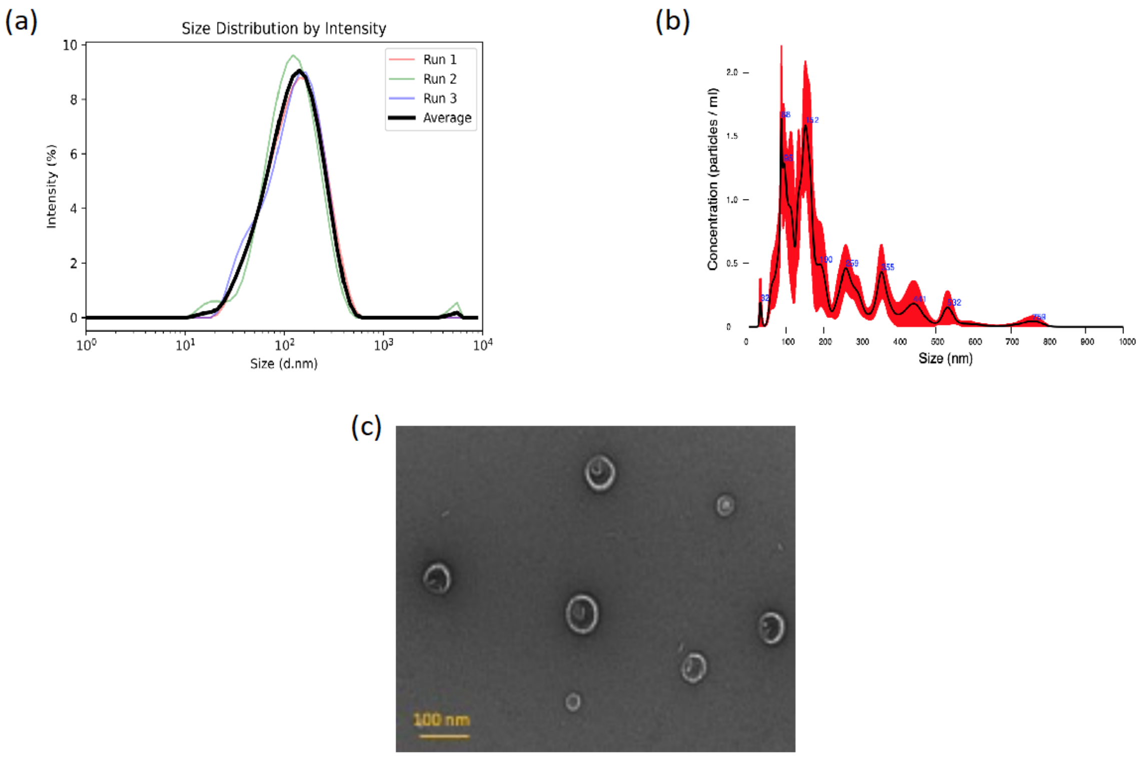

Figure S1: OMV characterisation. (a) DLS shows that the size of OMVs is in the expected 20-200 nm range (b) NTA shows more precise size mapping, with the major OMV size peaks being at 88,95 and 152 nm (c) TEM image showing the OMVs.

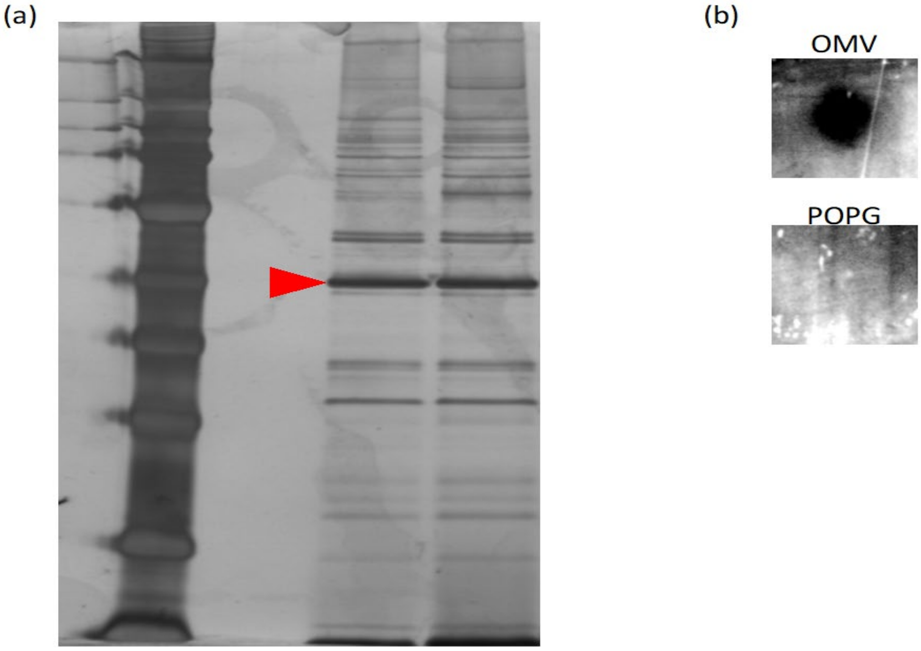

Figure S2: Characterisation of protein content of BL21 OMVs. (a) SDS-PAGE gel for two independently isolated BL21 OMV samples. A prominent band can be seen at ~40 kDa, which corresponds to the molecular weight of OmpC. (b) Dot blots of OMV and POPG samples against an OmpC marker. In the OMV sample, a dark spot can be clearly seen due to the secondary HRP antibody attaching to the OmpC primary antibody that has bound to OmpC proteins in the OMVs. The POPG sample shows no signal since the OmpC primary antibody is unable to bind.

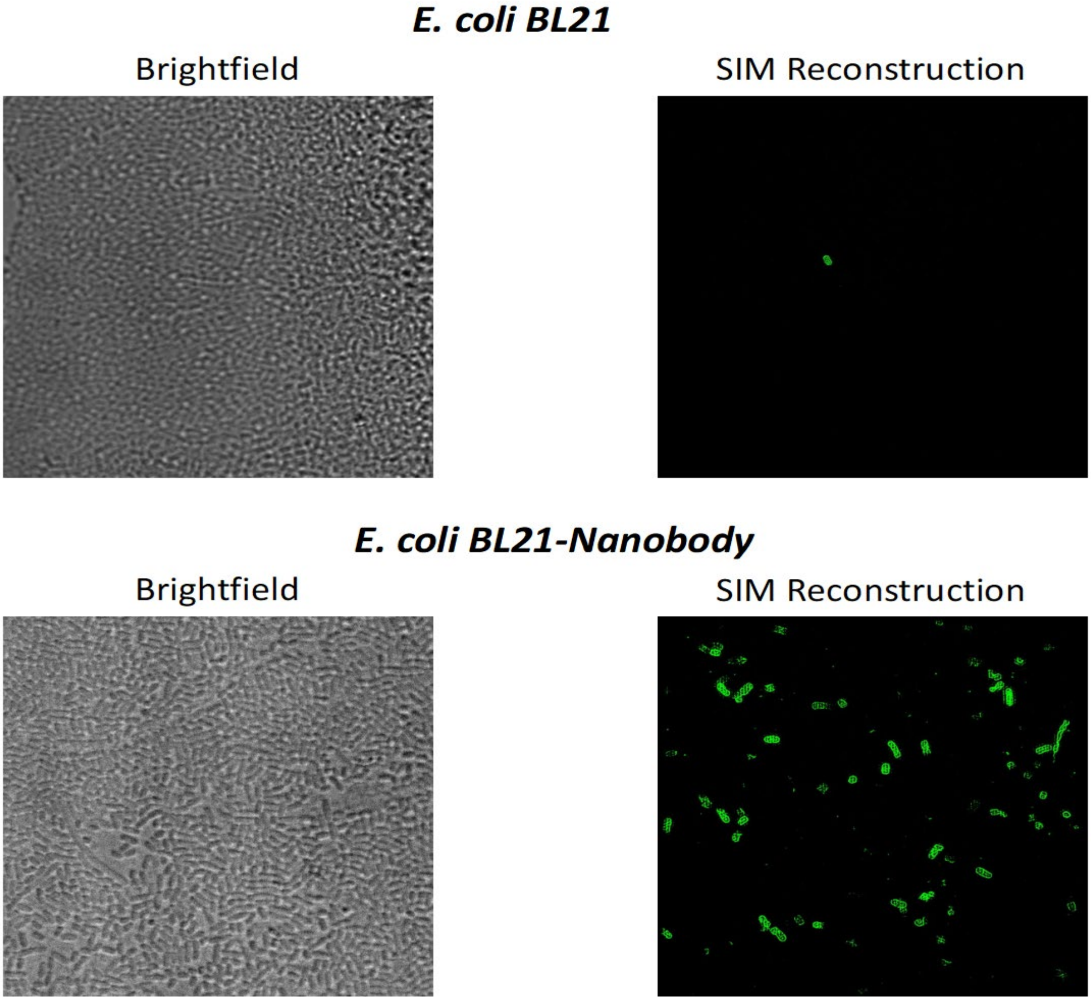

Figure S3: Whole cell *E. coli* transformed with LppOmpA-nanobody binding GFP assay. 0.01 mg/ml GFP solution is incubated with cells for 20 minutes at 30ºC, after which the cells are centrifuged, resuspended and washed in Tris buffer before imaging. In the BL21 sample, minimal interaction between the bacteria and GFP can be observed, while the BL21-nanobody sample shows a large number of bacteria decorated with GFP. Crucially, the signal arises from the surface of the cells, suggesting the GFP is binding to a surface protein as opposed to being internalised.

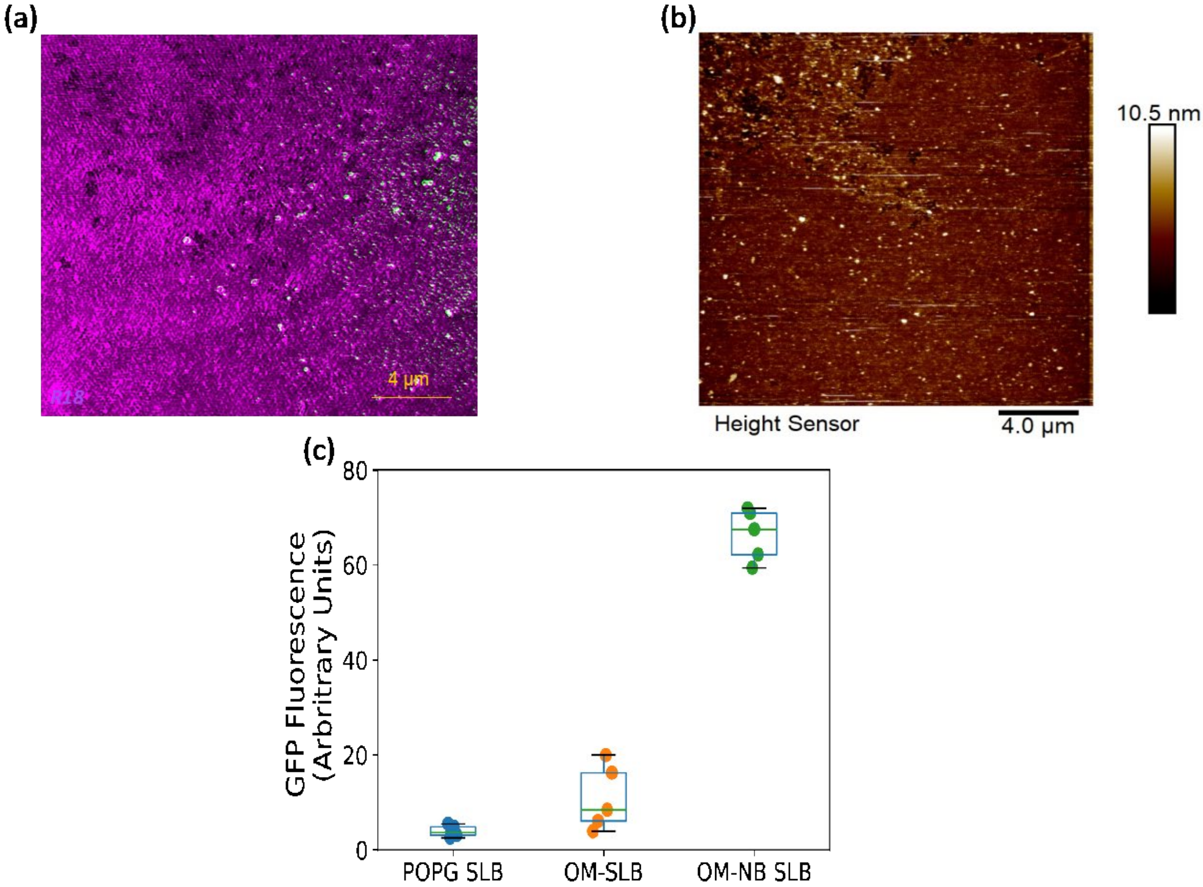

Figure S4: Correlative AFM/SIM images for POPG SLB incubated with GFP. (a) Reconstructed SIM image (b) AFM image. In both cases there is negligible GFP signal or binding. (c) Corrected total fluorescence (CTF) in the 488 nm range for the bacterial component region of OM-SLB vs OM-NB SLB vs representative region of POPG SLB. The bar chart clearly shows the increase fluorescence in the nanobody case compared to the two controls.

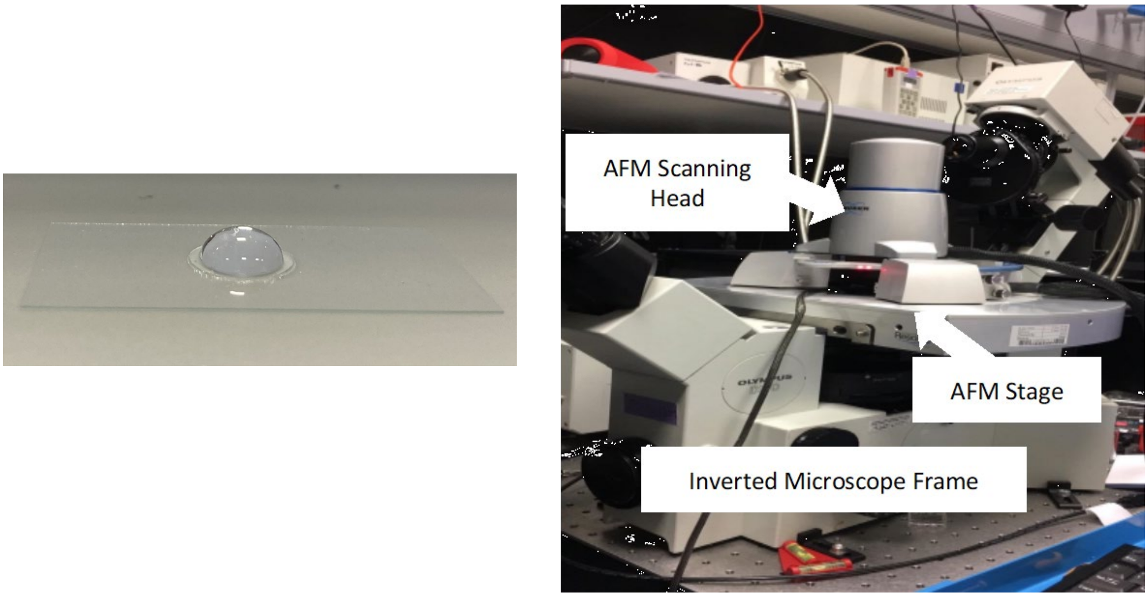

Figure S5: (left) Image of a representative sample of an SLB in buffer on a cover slip. (right) Annotated image of the correlative microscope setup.

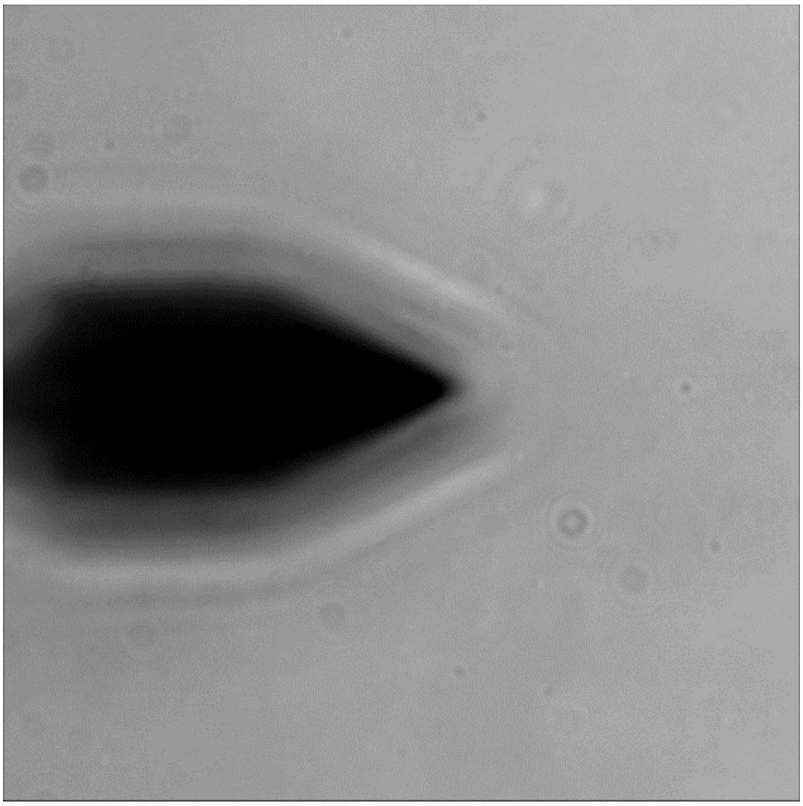

Figure S6: The AFM cantilever as imaged through the SIM microscope. The tip of the cantilever is positioned in the middle of the field of view of the SIM, ensuring the accuracy of the alignment of the fields of view of the two microscopes.

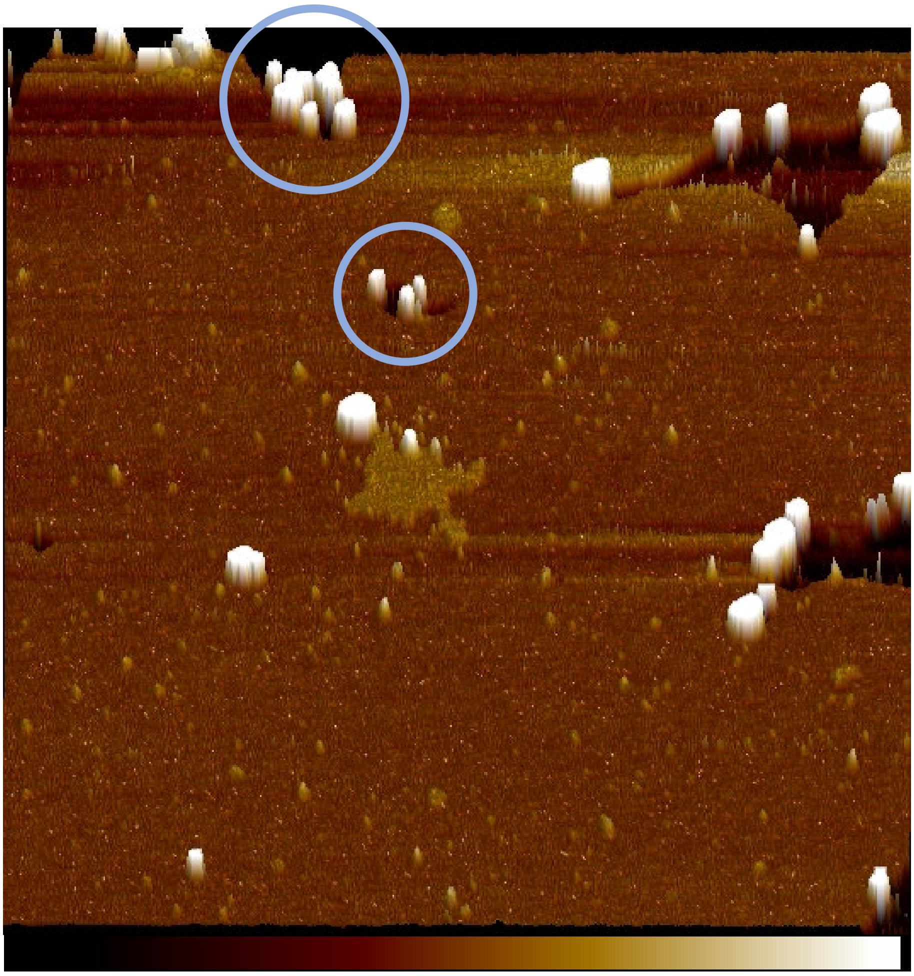

Figure S7: Atomic force microscopy scan of a 2 x 2 μm sample region. The synthetic POPG bilayer can be seen engulfing OMVs and causing them to rupture. Different numbers of OMVs are fused together and ruptured each time. Two such examples containing three and eight OMVs respectively are highlighted with blue circles
